# Effect of Mild Cognitive Impairment and Alzheimer Disease on Auditory Steady-State Responses

**DOI:** 10.18869/nirp.bcn.8.4.299

**Published:** 2017

**Authors:** Elaheh Shahmiri, Zahra Jafari, Maryam Noroozian, Azadeh Zendehbad, Hassan Haddadzadeh Niri, Ali Yoonessi

**Affiliations:** 1. Department of Neurosciences and Addiction Studies, School of Advanced Technologies in Medicine, Tehran University of Medical Sciences, Tehran, Iran.; 2. Department of Basic Sciences, School of Rehabilitation Sciences, Iran University of Medical Sciences, Tehran, Iran.; 3. Department of Psychiatry, School of Medicine, Tehran University of Medical Sciences, Tehran, Iran.; 4. Department of Geriatric Medicine, School of Medicine, Tehran University of Medical Sciences, Tehran, Iran.; 5. Department of Audiology, School of Rehabilitation Sciences, Iran University of Medical Sciences, Tehran, Iran.; 6. Research Center for Cognitive and Behavioral Sciences, School of Pharmacy, Tehran University of Medical Sciences, Tehran, Iran.

**Keywords:** Alzheimer disease, Auditory Steady-State Response (ASSR), Auditory ageing, Mild cognitive impairment, MCI detection

## Abstract

**Introduction:**

Mild Cognitive Impairment (MCI), a disorder of the elderly people, is difficult to diagnose and often progresses to Alzheimer Disease (AD). Temporal region is one of the initial areas, which gets impaired in the early stage of AD. Therefore, auditory cortical evoked potential could be a valuable neuromarker for detecting MCI and AD.

**Methods:**

In this study, the thresholds of Auditory Steady-State Response (ASSR) to 40 Hz and 80 Hz were compared between Alzheimer Disease (AD), MCI, and control groups. A total of 42 patients (12 with AD, 15 with MCI, and 15 elderly normal controls) were tested for ASSR. Hearing thresholds at 500, 1000, and 2000 Hz in both ears with modulation rates of 40 and 80 Hz were obtained.

**Results:**

Significant differences in normal subjects were observed in estimated ASSR thresholds with 2 modulation rates in 3 frequencies in both ears. However, the difference was significant only in 500 Hz in the MCI group, and no significant differences were observed in the AD group. In addition, significant differences were observed between the normal subjects and AD patients with regard to the estimated ASSR thresholds with 2 modulation rates and 3 frequencies in both ears. A significant difference was observed between the normal and MCI groups at 2000 Hz, too. An increase in estimated 40 Hz ASSR thresholds in patients with AD and MCI suggests neural changes in auditory cortex compared to that in normal ageing.

**Conclusion:**

Auditory threshold estimation with low and high modulation rates by ASSR test could be a potentially helpful test for detecting cognitive impairment.

## 1. Introduction

Mild Cognitive Impairment (MCI) is a disorder of the elderly, characterised by one or more cognitive deficits disproporionate to the person’s age and level of education (Golob, Irimajiri, & Starr, 2007; Irimajiri, Golob, & Starr, 2005). The impairments are observerd in various cognitive domains such as episodic memory, executive function, attention, language, and visuospational skills (Albert et al., 2011). Alzhiemer Disease (AD) is a gradually progressive disease with three phases: asymptomatic, symptomatic predementia, and symptomatic dementia. It is difficult for clinicians to identify transitions among these phases and differences between patients with early AD and the normal elderly. MCI seems to be symptomatic predementia in which subtle cognitive impairments in addition to mild structural changes similar to AD are observed. MCI should be clinically and cognitively evaluated (Bischkopf, Busse, & Angermeyer, 2002).

Identifying patients in the MCI phase is critical because interventions may help prevent progression to AD. Different methods have been used over the years to diagnose symptomatic pre-dementia. Unfortunately, none have been validated to detect MCI until now (Chertkow et al., 2009). Many psychometric tools have been designed to screen for MCI (Bischkopf et al., 2002), such as the Montreal Cognitive Assessment (MoCA) (Chertkow et al., 2009), Global Deterioration Scale (GDS) (Bischkopf et al., 2002), and Mini-Mental State Examination (MMSE) (Chertkow et al., 2009). Although these brief questionnaires are useful and broadly applied, they have low sensitivity to detect symptomatic predementia (Chertkow et al., 2009), which might be inefficient for diagnosing MCI (Bischkopf et al., 2002).

Biomarkers such as CSF amyloid β42, CSF tau/phosphorylated-tau, ratio of CSF tau/amyloidβ 42 and neural injury biomarkers for detecting symptomatic predementia have also been investigated. Structural imagings such as MRI and CT scan have been investigated over the years for identifying early signs of AD. Atrophy of cortical regions can be detected and may be predictive of MCI and AD. However, there is not sufficient evidence for their sensitivity and specificity (Chertkow et al., 2009). Electroencephalography (EEG) has also been used as a neuro-marker for detecting MCI and AD. Gamma band oscillations is reported to be increased in AD compared to MCI patients (Van Deursen, Vuurman, Verhey, van Kranen-Mastenbroek, & Riedel, 2008).

Temporal region is one of the initial areas imapaired in the AD (Van Deursen, Vuurman, van Kranen-Mastenbroek, Verhey, & Riedel, 2011). Therefore, auditory cortical evoked potential could be a valuable marker for detecting MCI and other neurodegenerative processes. Irimajiri et al. (2005) studied auditory long latency cortical potential for p50 amplitude. They reported larger P50 amplitude in MCI patients that reflect the presence of neurodegenerative pathology in MCI patients. These patients also exhibited enhanced N100 amplitude at slow stimulus rates but are similar to normal elderly people on fast rate (Irimajiri et al., 2005).

Fast neural oscillation is a repetitive and synchronized neural firing that cause integration of neural circuits in cognitive processes. The 40-Hz Steady-State Response (SSR) evoked by auditory stimulations is a method for evaluating these neural oscillation in the brain measured by magnetoencephalography (MEG) and EEG. A MEG study demonstrated that the 40-Hz Steady-State Response power in patients with AD was higher because of decreased cortical inhibition (Osipova, Pekkonen, & Ahveninen, 2006). Another EEG study evaluated the 40-Hz rhythm characteristic of MCI patients. Although 40-Hz power increases by disease progression, its usefulness in predicting the progression of MCI to AD is unclear (Van Deursen et al., 2011).

Sensory measures such as hearing evaluation are appropriate predictors of cognitive functions (Martini, Castiglione, Bovo, Vallesi, & Gabelli, 2015). Auditory Steady-State Responses (ASSR) is one of the methods of obtaining a pure tone threshold based on frequency and appears useful for estimating the hearing thresholds of passive patients. Recent studies have used ASSR to estimate hearing thresholds or to evaluate neural dyssynchrony, such as auditory neuropathy. There is, however, no study using this test in patients with AD and MCI. In our study, auditory evoked potential was recorded from scalp by far field electrodes and was evoked by repitition amplitude/frequency modulated tones or clicks (Jafari, Malayeri, Ashayeri, & Farahani, 2009). ASSR with 80 Hz modulation rate is suggested to be obtained from brainstem activitation; while 40 Hz modulation rate possibly originates from the temporal cortex (Herdman et al., 2002; Usubuchi et al., 2014).

It is suggested that 40-Hz ASSR repsonses reflect early sensory processing (Van Deursen et al., 2011). Evidence also suggests that sensory processing is one of the earliest impaired functions in AD. Therefore, we investigated 40-Hz ASSR threshold in different frequencies, potentially acquired from the cortex (Usubuchi et al., 2014) and compared them with 80-Hz ASSR thresholds potentially obtained from lower brain regions (Herdman et al., 2002) in three groups. We compared the thresholds of the two modulation rates to identify whether their ASSR thresholds differ among different frequencies during neurodegenerative stages. Our hypothesis is that the 40-Hz stimulation threshold is higher in patients with AD than in those with MCI and normal controls.

## 2. Methods

### 2.1. Subjects

A total of 42 age-matched patients, including patients with AD (n=12), MCI (n=15), and control group (n=15) participated in this study. All were referred from the Memory Clinic at Roozbeh Hospital affiliated to Tehran University of Medical Sciences. Their demographic information is presented in Table 1. The aim of the study were explained to all participants and their consent was obtained. Inclusion criteria for normal participants were absence of history of head trauma, stroke, substance abuse, vascular disorders, and neurological disorder (Hudon, Belleville, & Gauthier, 2009; Van Deursen et al., 2008). Patients with MCI underwent neurological and psychiatric examinations, MRI and cognitive evaluation using MMSE (Irimajiri et al., 2005; Van Deursen et al., 2011). Activities of daily life were evaluated using clinical interviews and functional assessment staging (FAST) scale (Sclan & Reisberg, 1992).

**Table 1:** Demographic information.

	**Control Group**	**MCI**	**AD**
n	15	15	12
Age (year), mean±SD	68.5±3.3	67.3±5.9	70.1±5.8
Gender (male/female)	7/8	4/11	3/9

It was important that patients with MCI could perform all activities of daily living well and independently (Sclan & Reisberg, 1992). The main inclusion criteria for patients with AD were having evaluated neurologically and neuropsychologically by neurologists, using ADRDA/NINCDS and DSM-V criteria (Van Deursen et al., 2011; Van Deursen et al., 2008). This group exhibited impaiments in daily activites, assessed by clinical interview and the functional assessment staging test (FAST) scale (Sclan & Reisberg, 1992). Finally, diagnosis of MCI and AD was made by biweekly visits of neurologists at the Memory Clinic of Roozbeh Hospital. Our study was cross-sectional study and type of sampling was convenience method.

### 2.2. Audiometry

Pure tone audiometry was measured with the Hughson-Westlake method (Jafari et al., 2009) at 3 frequencies (500, 1000, and 2000 Hz) binaurally in all cases. This evaluation was performed with a calibrated GSI audiometer in an acoustic chamber. It was considered that all groups were in a normal hearing threshold under 25 dB Hearing Level (HL) to control for the effect of hearing loss on our study (Ferraro, Durrant, & Katz, 1994; Martines, Bentivegna, Martines, Sciacca, & Martinciglio, 2010a, 2010b).

### 2.3. Electrophysiological recordings

ASSR was recorded using Eclips (Intra-acoustic company) at 2 modulation rates; 40 Hz and 80 Hz. At the beginning, the procedure of ASSR evaluation was explained to all participants and they were asked to lie on the bed in a supine position, try to relax, close their eyes, and remain awake during the test. We gave them a short rest between each rate modulation test.

The test was conducted in a sound attenuation chamber, which was shielded electrically. After skin preparation, 4 electrodes were placed on each mastoid, one on the vertex and one on the low forehead as the ground connection. Electrode impedances were kept below 5 kΩ. Earphones were inserted into ear canals. Air conduction ASSR was recorded at 3 carrier frequencies; 500, 1000, 2000 separately, for both ears and at 40 and 80 modulation rates. For finding threshold for each frequency at 40 or 80 Hz stimulation, the initial level was 40 dB HL. This was decreased by 10 dB and then increased by 5 dB, consistent with the modified Hughson-Westlake method. We detected the threshold at minimum response levels in each frequency in both ears and 2 modulation rates of 40 and 80 Hz, separately. Both amplitude and frequency modulation were applied for stimulus at depths of 80 and 20, respectively (Jafari et al., 2009; Panahi, Jafari, & Hasani, 2014).

### 2.4. Data analysis

ANOVA test was used to compare differences among ASSR threshold at 40 and 80 Hz at each frequency among 3 groups. To examine the differences between ASSR threshold at 40 and 80 Hz at each frequency separately, each group was analysed using paired t test. P value was considered significant at the level of 0.05.

## 3. Results

### 3.1. ASSR rate in 2 modulation rates

**Figure 1:**
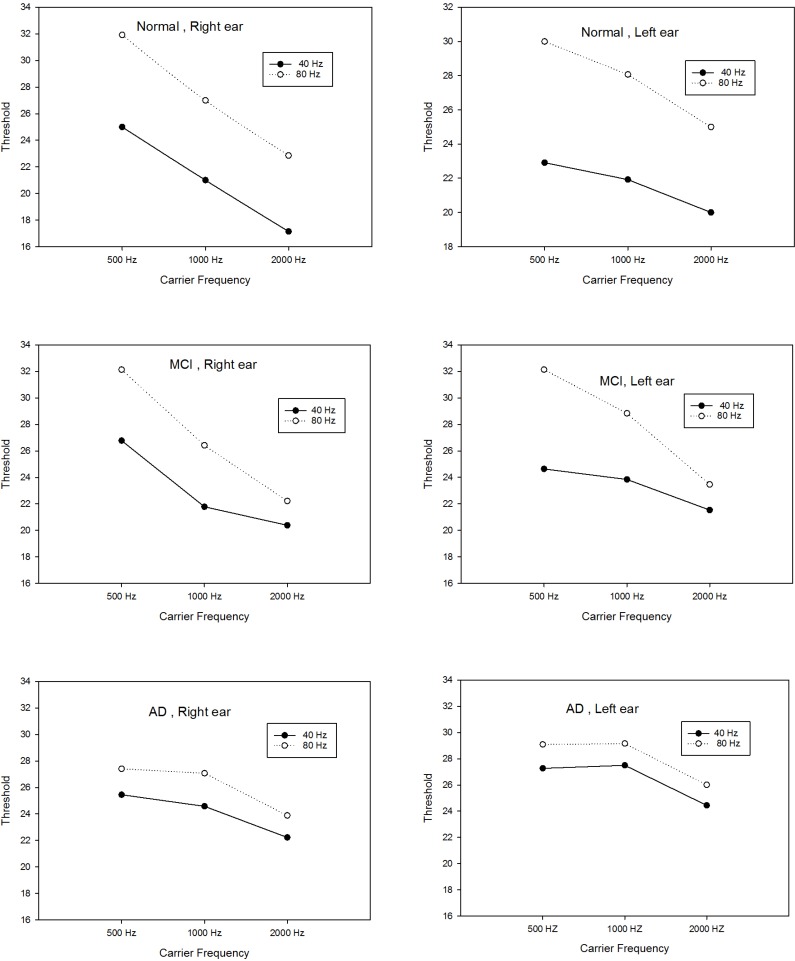
Plotting 40-Hz and 80-Hz ASSR thresholds for 500, 1000, and 2000 Hz for each group and ear.

Table 2 and Figure 1 present the means and standard deviations of the ASSR thresholds in 2 modulation rates and 3 frequencies in each group. With reagrd to estimated ASSR thresholds, there was significant difference between the two modulation rates (40 and 80 Hz) for 3 frequencies in both ears in the normal group and for 500 Hz frequency in the MCI group, but there was no significant difference in ASSR thresholds in 2 stimulation rates for 1000 and 2000 Hz in the MCI group. No significant differences in the ASSR thresholds of the 2 modulation rates (40 and 80 Hz) were observed among the 3 carrier frequencies in both ears of the patients in AD group.

**Table 2:** The means and standard deviation of the estimated ASSR threshold at 2 modulation rates, in 3 frequencies, in each studied groups and both ears.

	**ASSR**	**RE. Threshold (dB)**					**LE. Threshold (dB)**				

		**40 Hz**		**80 Hz**		**P**	**40 Hz**		**80 Hz**		**P**
	
		**Mean**	**SD**	**Mean**	**SD**		**Mean**	**SD**	**Mean**	**SD**	
AD	500 Hz	25.45	7.22	27.41	9.82	0.594	27.27	8.47	29.09	8.31	0.617
	1000 Hz	24.58	10.54	27.08	9.64	0.551	27.50	6.21	29.16	6.68	0.534
	2000 Hz	22.22	10.34	23.88	10.54	0.739	24.44	8.45	26.01	6.66	0.667
MCI	500 Hz	26.78	7.49	32.14	8.25	0.058	24.64	7.45	32.14	6.99	0.011
	1000 Hz	21.78	7.49	26.42	10.08	0.178	23.84	10.83	28.84	11.02	0.155
	2000 Hz	20.38	6.91	22.22	10.34	0.474	21.53	7.46	23.46	8.51	0.546
NC	500 Hz	25.00	4.56	31.92	4.80	0.001	22.91	6.89	30.00	7.38	0.024
	1000 Hz	20.50	7.12	27.00	7.74	0.036	21.92	7.51	28.07	7.51	0.047
	2000 Hz	17.14	7.77	23.50	8.48	0.055	20.00	6.03	26.50	5.64	0.048

Abbreviations: AD: Alzheimer Disease, ASSR: Auditory Steady-State Response, LE: Left Ear, MCI: Mild Cognitive Impairment, MR: Modulation Rate, NC: Normal Control, RE: Right Ear, SD :Standard Deviation.

### 3.2. Comparison of ASSR thresholds difference between normal, MCI, and AD groups with regard to 2 modulation rates

Table 3 summarizes the least significant difference (LSD) post hoc test for multiple comparisons of group means in ASSR thresholds difference for the 2 modulation rates at each frequency (500, 1000, and 2000 Hz) in both ears. The LSD post hoc test revealed significant differences between the normal and AD group with reagrd to ASSR thresholds difference in 2 modulation rates (40 and 80 Hz) at 3 frequencies in both ears.

**Table 3:** Results of the LSD post hoc test for multiple comparisons of group means in ASSR thresholds difference in two modulation rates in each frequency in the both ears.

**ASSR**		**Right Ear**						**Left Ear**					

		**MR Difference**			**P**			**MR Difference**			**P**		

		**AD**	**MCI**	**NC**	**AD**	**MCI**	**NC**	**AD**	**MCI**	**NC**	**AD**	**MCI**	**NC**
500 Hz	AD	-	−4.43	−4.96	-	0.022	0.016	-	−5.68	−5.27	-	≤0.001	≤0.001
	MCI	4.43	-	−0.53	0.022	-	0.210	5.68	-	0.61	≤0.001	-	0.597
	NC	4.96	0.53	-	0.016	0.210	-	5.27	−0.61	-	≤0.001	0.597	-
1000 Hz	AD	-	−2.14	−4.00	-	0.163	0.024	-	−3.34	−4.49	-	0.1.41	0.004
	MCI	2.14	-	−1.86	0.163	-	0.347	3.34	-	−1.15	0.141	-	0.308
	NC	4.00	1.86	-	0.024	0.347	-	4.49	1.15	-	0.004	0.308	-
2000 Hz	AD	-	−0.18	−4.70	-	0.863	0.009	-	−0.36	−4.93	-	0.581	0.006
	MCI	0.18	-	−4.52	0.863	-	0.007	0.36	-	−4.57	0.581	-	0.009
	NC	4.70	4.52	-	0.009	0.007	-	4.93	4.57	-	0.006	0.009	-

Abbreviations: AD: Alzheimer Disease, ASSR: Auditory Steady State Response, LE: Left Ear, MCI: Mild Cognitive Impairment, MR: Modulation Rate, NC: Normal Control, RE: Right Ear,

In addition, the analysis revealed significant differences between the normal and MCI group with regard to ASSR thresholds difference in 2 modulation rates at 2000 Hz and between the AD and MCI group regarding ASSR thresholds difference in 2 modulation rates at 500 Hz in both ears.

## 4. Discussion

### 4.1. The ASSR thresholds in two modulation rates in each of the studied group

The present study showed significant differences in estimated ASSR thresholds in 2 modulation rates (40 and 80 Hz) for 3 frequencies in both ears in the normal elderly group. In this group, 40-Hz ASSR threshold was higher than 80-Hz ASSR threshold in 500, 1000 and 2000 Hz. Previously, comparison of 2 modulation rates of 40- and 80-Hz ASSR threshold in normal adults had shown that 40-Hz ASSR threshold is significantly better than 80-Hz ASSR threshold (Picton, John, Purcell, & Plourde, 2003; Van Maanen & Stapells, 2005). Evidence obtained from fMRI data has shown that during 40 Hz modulation rate, blood flow increases in auditory cortex and upper brainstem (Picton et al., 2003; Plourde et al., 2008; Purdon et al., 2004). In another study using animal model, 40-Hz ASSR was shown to be generated from the auditory cortex, in the thalamus and brainstem, especially the inferior colliculus. Thus, the regions between the brainstem and auditory cortex are excited by low modulation rates (40 Hz) and because of greater neuronal connection in these regions, a better ASSR threshold was obtained, whereas the main generator of high modulation rate (80 Hz) is suggested to be the brainstem, with less neural connection relative to the cortical region (Herdman et al., 2002; Plourde et al., 2008).Despite significant differences in 2 modulation rates of ASSR threshold in normal elderly people, in AD group, the 40-Hz ASSR thresholds were approximately similar to 80-Hz ASSR thresholds for the 3 frequencies in both ears. Thus, no significant differences were discovered in estimated ASSR thresholds in 2 stimulation rates (40 and 80 Hz) in this group. Neuropathological studies indicate that the temporal cortex is one of the first regions affected by AD (Braak & Braak, 1995); thus the lack of difference between thresholds of these 2 modulation rates might be related to the insufficient cortical response to 40 Hz stimulation rate in this group (Van Deursen et al., 2011). Another study using magne-toencephalography reported that power of 40-Hz ASSR was higher in AD group than in controls, owing to decreased cortical inhibition in temporal area in the AD group (Osipova et al., 2006).

Additionally, in a study on 40-Hz Steady-State Response in AD and MCI patients, electroencephalogram records revealed that the effect of 40-Hz ASSR in AD is higher than in MCI and controls. This study suggestsed that 40-Hz steady-state power enhancement depends on severity of cortical impairment (Herrmann, Munk, & Engel, 2004; Van Deursen et al., 2011). The current findings are consistent with those of previous studies in terms of auditory cortical impairment affecting 40-Hz Steady-State Response in AD.

Significant differences in estimated ASSR thresholds emerged between the two stimulation rates (40 and 80 Hz) at 500 Hz frequency in both ears in the MCI group. There was no significant difference in 1000 and 2000 Hz frequencies in ASSR thresholds of 2 modulation rates, similar to that in AD group. In these frequencies, 40-Hz ASSR threshold increased and was closer to 80 Hz that might be due to auditory cortical activity impairment. Similar to our findings, in another EEG study on 40-Hz Steady-State Response (SSR) in MCI and AD, results indicated that power of 40 Hz SSR in the MCI group was lower than in patients with AD but higher than those in normal controls, owing to impaired cortical inhibition in the early stages of AD in temporal region (Osipova et al., 2006; Van Deursen et al., 2011).

According to our findings, the degree of cortical impairment in MCI was between normal elderly people and patients with AD, consistent with previous EEG studies (Van Deursen et al., 2011). An auditory long-latency cortical potential study on MCI patients has shown enhanced amplitude and delayed latency of P50 in the MCI group relative to the normal elderly group (Cancelli et al., 2006; Jessen et al., 2001). P50 reflects neural auditory cortical activity and these differences between the MCI and normal group on P50 amplitude reflect an increased response in auditory cortical neuronal activity to afferent input, as well as changes in the auditory cortex of MCI patients relative to the control group (Irimajiri et al., 2005; Liegeois-Chauvel, Musolino, Badier, Marquis, & Chauvel, 1994), which is consistent with our study. One of the interesting findings in the current study in MCI group is that 40-Hz ASSR thresholds increase (worsen) at higher frequencies. This means that, in the MCI group, 40-Hz ASSR threshold on moderate (1000 Hz) and higher (2000 Hz) frequencies are more affected than low frequency whereas in AD group more frequency regions are affected and revealed 40-Hz ASSR threshold increases (worse) in all frequencies, relative to the MCI and control groups. This is probably due to neurodegeneration in patients with AD being more excessive in the auditory cortex than in MCI group (Van Deursen et al., 2011).

### 4.2. The ASSR thresholds difference in 2 modulation rates between 3 groups

Comparison of ASSR thresholds differences in 2 modulation rates of 40 and 80 Hz between 3 groups revealed significant differences between the normal and the AD groups at 500, 1000, and 2000 Hz, in both ears. Significant differences between these two groups was due to cortical responses at 40 Hz modulation rate in the normal group that showed better (lower) threshold than these cortical responses in the AD group. In another investigation using magnetoencephalography, significant differences in the power of 40 Hz auditory SSR between patients with AD and healthy elderly people was observed (Osipova et al., 2006; Roß, Borgmann, Draganova, Roberts, & Pantev, 2000). Another EEG study indicated that the power of 40-Hz SSR in the AD group was significantly higher than in the MCI and control groups (Van Deursen et al., 2011). These studies are consistent with our results.

In the current study, comparison of ASSR thresholds difference in two modulation rates (40 and 80 Hz) between normal and MCI groups showed significant differences at frequencies of 2000 Hz in both ears between these groups. This is probably due to insufficient cortical responses in 40 Hz stimulation rate at 2000 Hz frequency in MCI group. So higher vulnerability of higher frequency areas in the auditory cortex is revealed in cognitive impairment. Consistent with our study, another study showed differences in the MCI group in terms of power of 40-Hz SSR between AD and control groups (Van Deursen et al., 2011). Prior studies on 2 baseline auditory sensory and cognitive potential have shown that P50 amplitude was larger in an MCI than a control group at all stimulation rates (Irimajiri et al., 2005). An investigation of P300 as cognitive potential revealed that MCI exhibited higher P300 latency than controls group (Golob et al., 2007). The current results confirm those in this previous study.

This study had some limitations, too. For instance, we had considered 4000 Hz carrier frequency for assessment but according to our inclusion criteria we needed normal hearing threshold for rolling out hearing loss effect on our study. Whereas our clients were between 60–80 years old and most of them had 4000 Hz hearing loss because of presbycusis. In this reahrd, we had to omit 4000 Hz carrier frequency assessment from our study.

In conclusion, this study showed that ASSR test can distinguish patients with AD from MCI and controls, by comparing thresholds of two modulation rates, 40 and 80 Hz, in these group. In addition, the current study suggests that differences between 2 modulation rate thresholds that decrease with respect to frequency, might predict severity of cognitive impairment.
